# Brian: The Typographical Error that Brought Early Career Neuroscientists and Artists Together

**DOI:** 10.1371/journal.pbio.1001340

**Published:** 2012-06-05

**Authors:** Megan J. Dowie, Erin Forsyth, Leah Forsyth

**Affiliations:** 1 Centre for Brain Research, Faculty of Medical and Health Sciences, University of Auckland, Auckland, New Zealand; 2 The Busy Nice, Auckland, New Zealand

## Abstract

In the project “Do You Mind?” early career neuroscientists and artists collaborated to produce artworks for exhibition.

## Introduction

The divide between the arts and sciences is a relatively modern phenomenon. One can look at Renaissance collaborations between naturalists and artists, including the rich body of botanical art from the 1500s, as historical examples of such interdisciplinary collaborations [1]–[4]. But as both fields have evolved, it's not surprising that they have become more compartmentalised and culturally segregated. Today, art and science subjects are taught independently from an early age, the divisions often solidifying over time [5]. Indeed, it is a great generalisation to limit definitions to merely “science” and “art,” with so many distinct categories within each field.

**Figure 1:**
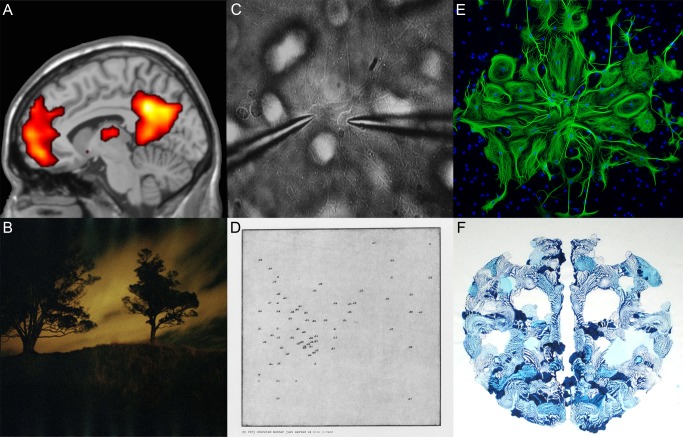
Examples of representative images produced by participating scientists in their research and corresponding artworks. (A) Reece Roberts analyses functional magnetic resonance imaging (fMRI) data from experiments investigating a core network of brain regions involved in both remembering the past and imagining the future. (B) In response Lia Kent MacKillop produced photographs referencing the associations made when remembering (or creating) places and experiences; *Untitled – 1/4 in series called Long Term*, Epson Premium Luster print from colour negative, 250×250 mm. (C) Juliette Cheyne uses electrophysiological techniques, including patch clamping, to record from individual neurons in culture to investigate their electrical properties and the neurobiological basis of memory. (D) In response Timothy Chapman made etchings that were essentially graph plots of mnemonic phrases common to scientific concepts, produced using a binary translator to turn the mnemonic phrase into numeric form. The title and image are therefore like two versions of the same information, referencing the physical neuron connections and the more ephemeral memories they create; *My very educated mother just served us nine pizzas* (detail), Etching on paper, first edition, 138×550 mm. (E) Renee Gordon works with stem cells that develop into mature brain cells under certain conditions (fluorescently labelled astrocytes are green and their nuclei are blue). (F) In response Tom Henry produced prints by pressing paint between surfaces to create semi-symmetric shapes, referencing brain hemispheres and regeneration of new cells; *Regeneration*, Acrylic and ink on paper, 400×250 mm. All artworks were produced in 2010, are not shown to scale, and are included here courtesy of the artist.

Projects that enlist scientists and artists to incorporate both perspectives have the potential to promote scientific research in the public arena, enrich the creative component of science, stimulate artists, and engage diverse communities in dialogue and discourse, while developing exposure for both fields. *Do You Mind?*, an art-science collaboration started by researchers at the University of Auckland Centre for Brain Research and the arts management business The Busy Nice, was initially inspired by the imagery produced within neuroscience as tools to start conversation—from functional magnetic resonance imaging (fMRI) scans of the human brain to the recording of electrical signals from brain cells in culture to fluorescent microscopy images of cells (Figure 1).

*Do You Mind?* paired early career neuroscientists at the Centre for Brain Research with newly established local artists. Collaborators started with the scientists' research, but were free to discuss any aspect of the brain and brain research. Artists then produced artworks for exhibition in response to this interaction and research.

The direct outcomes of *Do You Mind?* included a large-scale public exhibition and a publication documenting the project, with images and responses from all participants. Overall, developing *Do You Mind?* as a community project, using collaborative approaches, multi-media engagement, and documentation throughout the project, helped ensure high-profile media promotion. Anecdotal feedback from both the artists and scientists suggested that involvement had a positive effect upon their perspective and professional practice. The high level of public and media interest not only increased awareness of current neuroscience research at the Centre for Brain Research but also captivated fresh audiences for both research and art in Auckland.

## Building Art and Science Partnerships

Researchers were recruited internally in the Centre for Brain Research and the project was promoted to artists via a local creative website or by personal contact. Both early career scientists and newly established artists were encouraged to participate. Upon selection, participants attended an informal introductory evening. Participants then had 8 wk to produce artworks (with check-ins scheduled at 3 and 6 wk) and were required only to start with the scientist's research theme and to produce artwork through an organic process. Early on, project curators dubbed the project “Brian,” personifying it with a whimsical identity, resulting from a simple mistyping of the word “brain.”

(For a description of how the discussion was facilitated and what types of online resources were used, please see Text S1.)

## Results of Collaboration

The artworks and accompanying publication were exhibited in a launch event and over a 10-d period. The exhibition was staged independent of the University or an established gallery in order to set a neutral tone, ensuring accessibility to broad audiences.

**Figure 2:**
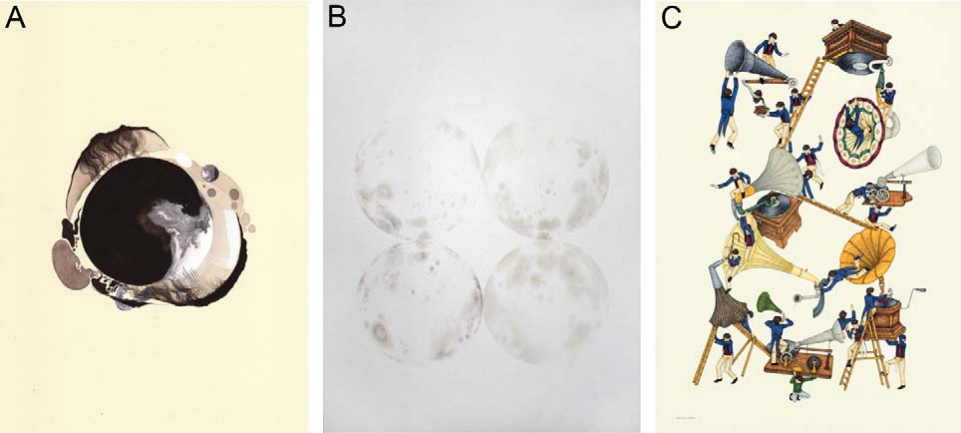
Examples of some of the artworks produced for *Do You Mind?* Examples here include works by (A) Henrietta Harris, in response to autoradiogram images from sections of human post-mortem brain tissue; *Autoradiograph*, Gouache on paper, 210×297 mm; (B) Aaron King-Cole, in response to methamphetamine addiction being investigated using DTI and MRI; *Phases of Acircadia–Lateral Descent I*, Watercolour on archival paper, 700×1,000 mm; and (C) Aleksandra Petrovic, in response to research into auditory attention training drew visual representations of tinnitus; *Untitled*, Pen, ink and pencil on paper, 500×700 mm. All artworks were produced in 2010, are not shown to scale, and are included here courtesy of the artist.

Artworks ranged from organic sculpture to contemporary watercolours, sound production to oil paintings. The research-based style of the project and unconventional theme enabled many artists to explore new mediums, and more than 40 artworks were submitted for display from the 15 partnerships (Figures 1 and 2). Research themes came broadly from across the Centre for Brain Research, including studies on methamphetamine addiction, perception of music and its correlation with movement, neural stem cell migration, tinnitus and attention, the brain's hemispheric laterality, and remembering the past/imagining the future (a brief summary of all 15 pairs is described in Table 1). The publication included summaries of the neuroscience research projects with samples of corresponding art, and all participants were asked to contribute a brief written response their involvement (the publication can be viewed on the blog at doyoumind.tumblr.com/publication). An informal evaluation of these texts demonstrates primarily positive feedback from a diversity of relationships (see Table S1).

**Table 1:** Brief summary of research covered by the neuroscientists, additional themes discussed, and the art medium and/or response chosen for use by the partnering artist.

Research Area (Theory, Techniques, Other Features Discussed)	Art Medium and Response
Clinical depression, identity, stem cells/neurogenesis, histology, stained brain tissue sections on glass slides	Charcoal portrait representing identity printed onto transparency, mounted freestanding and upright, with sectioned portions mimicking slides/coverslips
Auditory-motor associations during and after musical training, plasticity of the brain in the sensorimotor domain, recording using electroencephalography (EEG)	Large site-specific wall-mounted installation modelled upon key EEG recording locations on the skull, using vinyl cut and perspex cubes, including images of musical notation and boxers (demonstrating action)
Epigenetics in neurodegenerative diseases, human post-mortem brain tissue, microscopy (concept of scale)	Conceptual sculptures including a concrete plinth mimicking a gravestone, flag-pole with plastic banner and painted words and a watercolour painting representative of the human brain
Brain immune cells, microglia function, human post-mortem brain cells grown in culture, magnification (concept of scale), microscopy	Painting and digital manipulation, collage, with geometric shapes and references to size/scale including galaxies/space
Stroke and brain injury/repair, histology, identification of proteins in tissue sections	A series (6×10) of small delicate abstract watercolours mounted together
Neurodegeneration, stem cells, migration of cells, degeneration and regeneration, fluorescent imaging microscopy (Figure 1E)	Print making, pressing paint between surfaces to create random, natural forms in semi-symmetric shapes, alluding to the brains hemispheres and regeneration (Figure 1F)
Auditory mechanics and perception in autistic spectrum disorders, dichotic pitch, EEG recordings	Aural interactive sculpture using sounds specific to the research, sound installation activated when a circuit is completed by the viewer, referencing dichotic pitch
Tinnitus (phantom sounds) and auditory attention training, audiology, EEG recordings	Detailed pen and ink drawings, with features directly relevant to the research (e.g., people, oversized gramophones, musical instruments, tinnitus as a burden) (Figure 2C)
Memory formation, brain cells in culture, electrophysiology, recording of electrical signals from cells, synapses and how neurons communicate (Figure 1C)	Prints made by etching, images mimicking “connect the dots” pictures, with references to mnemonics used in learning of scientific concepts (Figure 1D)
Amblyopia (lazy eye) and visual perception, visual evaluation equipment, vision training	Multimedia video installation using research equipment and art/science participants, referencing the researchers visual training tests, printmaking
Memory and imagination investigated using functional magnetic resonance imaging (fMRI), which uses blood flow as a measure of brain activity (Figure 1A)	Photographs primarily of landscapes taken while travelling, in reference to the associations made when remembering (or creating) places and experiences (Figure 1B)
Memory and imagination, creativity and art, evolutionary aspects, investigated using fMRI	Conceptual painting in monochromatic tones and the transcript of an interview with a psychic
Methamphetamine use and evaluation of potential pharmacological treatments for addiction, brain imaging including diffusion tensor imaging (DTI) and fMRI	Abstract circular watercolours, influenced by concepts associated with the movement of water and drug-induced behaviours, referencing the moon (lunar) and madness (“lunacy”) (Figure 2B)
Brain asymmetries, differences in structure and function between the hemispheres, fMRI, stereotype of a (young woman) scientist	Large-scale portrait in oils, representing the scientist (with a model stand-in), investigating the role/image of female scientists in popular culture, identity, character
Post-mortem human brain tissue, microscopy, autoradiography, neurodegeneration, histology	Abstract biological paintings, influenced by changes seen when focusing on a light microscope and free-hand responses to autoradiogram film images (Figure 2A)

Those partnerships for which images have been included in Figure 1 or 2 have been noted in parentheses.

## Lessons from the Collaboration

Public engagement in scientific issues is vital. Indeed, with social questions inherent to brain research, the field of neuroscience has further responsibilities to facilitate dialogue [6]. Approaching this issue through collaboration and interaction with a creative community, benefiting both parties, helps encourage non-scientists to engage with scientific research. Formalised interactions between artists and scientists are relatively new, with exciting and stimulating results to date [7]–[11], and internationally there is increasing recognition, support, and funding for such interactions [12]–[14].

Vital features making *Do You Mind?* a successful cross-disciplinary collaboration included the interaction between paired artists and scientists, the freedom to mutually explore ideas, and the challenge of a loose conceptual framework. A relevant feature of this project is the embedded interest from the Centre for Brain Research, as a high proportion of science-art projects are artist-initiated or led [15],[16]. For scientists, *Do You Mind?* offered increased science communication, promotion of research and science outcomes to society through media exposure, and engagement with different communities, including younger generations. The project also encouraged creative thinking as scientists saw their own and others' research in a different light and became more aware of serendipitous opportunities in their results. For artists, *Do You Mind?* offered newly established artists public exposure as well as access to a world they may not normally inhabit, with the challenge of an unconventional theme and short timeframe. The experience of three of the artists was captured in a video produced by The Busy Nice for the Science Communicators Association of New Zealand (SCANZ) annual conference 2011 (http://www.youtube.com/watch?v=YFH9b56aBL0&feature=player_embedded).

Through the initiative, the artists and scientists realised they have much in common. Artists and scientists are similarly interested in understanding nature, order, and function, and ask questions in similar ways, developing hypotheses, experimenting, and testing ideas. Of course, we present our conclusions in different ways. Scientific research is definite, unambiguous, specialised, and intentional. In contrast, artists are speculative and explicitly open themselves to critique, inviting unique opinions and interpretations, sometimes even intending to challenge an audience. Narratives exploring science and art, and the disjunct between them, may lead to a more holistic approach to their research by both artists and scientists.

Projects like *Do You Mind?* give artists a wider range of enquiry while encouraging scientists to be more comfortable with uncertainty. As Robert Sapolsky so aptly stated, “science is not meant to cure us of mystery, but to reinvent and reinvigorate it” [17]. Despite all the ambitious objectives possible from art-science collaborations, the most unpretentious and rewarding outcome of *Do You Mind?* was the initiation of discussions, across communities and disciplines. Aside from the affectionate naming of the project as the unassuming “Brian,” Mei Cooper provided an insightful artistic response to Pritika Narayan's research into epigenetic changes occurring in neurodegenerative diseases. Cooper made a metal pole flaunting a long transparent plastic banner with words painted in silver saying: “The innumerable task of generating problems to solve tomorrow.” The definition of research, perhaps?

## Supporting Information

Table S1 Artists and scientists respond in writing (excerpts).
(DOCX)

Text S1 Facilitation of discussion and online resources used.
(DOCX)

## Abbreviations

DTI: diffusion tensor imaging
EEG: electroencephalography
fMRI: functional magnetic resonance imaging
