# Supplementary material for: Brian: The Typographical Error that Brought Early Career Neuroscientists and Artists Together
Source: PLoS Biol. 2012 Jun 5;10(6):e1001340. doi: 10.1371/journal.pbio.1001340 (PMC3367990; doi:10.1371/journal.pbio.1001340)
Supplement: Table S1 — Artists and scientists respond in writing (excerpts). (DOCX) [file pbio.1001340.s001.docx]

| Tom Henry (artist): “One thing that strikes me about Renee‘s research is the idea of growth and regeneration…Another fascinating element is the images she produces from the microscopic brain cells, evoking everything from star-like galaxies to seaweed forests.” (Fig 1 F) |
| --- |
| Renee Gordon (scientist): “I’ve learnt about art practices and realised that even though art and science are completely different disciplines, there are similarities between the optimisation of artwork and scientific experiments.” (Fig 1 E) |
| Aaron King-Cole (artist): “My thoughts continued to return to a remark Jo made early on about methamphetamines influencing the movement of blood and water in the brain. This observation struck me as something comparable to tidal movement of water caused by the Moon. As other parallels…became apparent, their relationship became more substantial and significant…My objective became to articulate, in essence, the two subjects simultaneously.” (Fig 2 B) |
| Joanne Lin (scientist): “[Aaron] came up with concepts and linked them in ways I never would have thought of. It was fun showing Aaron around…these things are so ordinary to me…but it was great that they were so interesting to him. I had the same feeling when I got the opportunity to see Aaron’s workspace...The whole project was so inspirational and worthwhile.” |
| Sophie Bannan (artist): “Rather than simply using the research I had access to, it was important for me to actively utilise it, looking for commonalities in both our fields of work in order to accurately represent them and their points of intersection. To view lazy eye research through a performative gaze lends itself to a dissection of process rather than result…with attention to the actions taking place in the lab (as opposed to the results or statistics they produce), it was fundamental that my researcher played a role in the actual making of the work, the product of which I regard to be a successful collaboration.” |
| Lucy Goodman (scientist): “Together a vision scientist and an artist with vision captured a unique and unimaginable image of science. *Do You Mind?* allowed me to step back and view research from a different viewpoint.” |
| Alexander Hoyles (artist): “I found that discussion/observation of the process of psychological testing was deeply interesting and also found myself fascinated with notions of unprocessed stimuli.” |
| Veema Lodhia (scientist): “Alex was able to take various elements of each lab visit to create his artwork…Alex’s artwork has captured the essence of the project perfectly”. |

Table S1: Artists and Scientists Respond in Writing [excerpts]. Some examples of the feedback from participants in *Do You Mind?* regarding their involvement, as quoted in the project publication. Participants often commented upon the positive nature of the interactions and developed ideas through collaborative investigations.
