# Supplementary material for: Brian: The Typographical Error that Brought Early Career Neuroscientists and Artists Together
Source: PLoS Biol. 2012 Jun 5;10(6):e1001340. doi: 10.1371/journal.pbio.1001340 (PMC3367990; doi:10.1371/journal.pbio.1001340)
Supplement: Text S1 — Facilitation of discussion and online resources used. (DOCX) [file pbio.1001340.s002.docx]

Facilitation of Discussion and Online Resources Used

Researchers were encouraged to discuss their theory (e.g. disease of interest, protein, signalling system, brain region), techniques and materials (e.g. magnetic resonance imaging, immunohistochemistry, radioactive compounds, cell culture) though digression to other brain-related discussions was allowed (e.g. research philosophies, scientific method, creativity, memory, identity, stereotypes, animal research, neurogenesis/neurodegeneration).

Online resources, including a customised private networking Ning site ([doyoumind.ning.com](http://www.doyoumind.ning.com)), facilitated participant networking, engagement and interaction, which was essential to the collective nature of this group project. Project curators posted updates regarding the exhibition, publication and media, and ran a *Do You Mind?* blog ([doyoumind.tumblr.com](http://www.doyoumind.tumblr.com)), which became crucial to documenting and promoting the event, recording site visits to art studios and research laboratories/clinics and supporting participants. The Centre for Brain Research website also hosted a specific *Do You Mind?* page ([cbr.auckland.ac.nz](http://www.fmhs.auckland.ac.nz/faculty/cbr/events/doyoumind.aspx)), which evolved with the project.
